# Announcement: Fourth International Symposium on Metal Ions in Biology And Medicine

**DOI:** 10.1155/MBD.1995.191

**Published:** 1995


					FOURTH INTERNATIONAL SYMPOSIUM ON METAL
IONS IN BIOLOGY AND MEDICINE
Tarragona / Barcelona, Catalonia, Spain
May 19-22, 1996
You are cordially invited to participate in the 4th International Symposium on Metal Ions in Biology
and Medicine, which will take place in Tarragona, Spain,19-22 May 1996.
ORGANIZING COMMITTEE
Domingo J L
Corbella J, Llobet JM, Collery Ph
G6mez M
Cannata J, Escanero JF, Hernandez A
Chairman
Vice-chairmen
General secretary
Members
LOCAL COMMITTEE
Bells M
De la Torre A
Paternain JL
Schuhmacher M
Colomina T
Folch J
S&nchez DJ
SCIENTIFIC COMMITTEE
Alfrey AC (USA)
Anghileri LJ (France)
Anke M (Germany)
Aposhian HV (USA)
Badawi AF (Egypt)
Barckhaus RM (Germany)
Bartrons R (Spain)
Belliveau JF (USA)
Carreras J (Spain)
Crook J (USA)
Etienne JC (France)
Evans HL (USA)
Farre R (Spain)
Farzami B (Iran)
Gielen M (Belgium)
Guinovart JJ (Spain)
Grandjean P (Denmark)
Ikeda M (Japan)
Jones MM (USA)
Khassanova L (Russia)
Klaassen CD (USA)
Keen CL (USA)
Kusaka Y (Japan)
Lauwerys R (Belgium)
Littlefield NA (USA)
Manfait M (France)
Millart H (France)
Moreno V (Spain)
Nagy L (Hungary)
Nelson R (USA)
Nomiyama K (Japan)
Nriagu JO (USA)
Oehme FW (USA)
Polissiou M (Greece)
Prasad AS (USA)
Prasad SB (India)
Poirier LA (USA)
Robberecht H (Belgium)
Sabbioni E (Italy)
Sava G (Italy)
Schrauzer GN (USA)
Sherlock JC (UK)
Sunderman FW, Jr. (USA)
Suzuki KT (Japan)
Tajmir-Riahi HA (Canada)
Theophanides Th (Greece)
Waalkes M (USA)
Winkelman G (Germany)
de Wolff FA (The Netherlands)
Yokel R (USA)
Zhang ZX (China)
Zidek W (Germany)
191
SCIENTIFIC PROGRAM
The scientific program will include symposia, oral and poster presentations with discussion sessions
and debates.
SCIENTIFIC TOPICS
The following topics will be discussed in relation to essential metals, toxic metal ions, and metal
interactions:
Biological/molecular interactions
Chemotherapy
Aging
Nutrition
Carcinogenesis
Immunology
Epidemiology
Toxicology
Cardiovascular diseases
Pathogenicity of metal internal protheses (including dental)
Chemistry
Biochemistry
This Symposium will address the role of metal ions in areas of biochemical, biological, and
medical sciences. The meeting will be of practical usefulness to those in basic and applied
research, clinical scientists, and workers in allied medical fields, biochemists, toxicologists,
and microbiologists.
LANGUAGE
The congress will be held in English.
Simultaneous translation will not be provided.
PROCEEDINGS
Selected papers will be published as a book by John Libbey Eurotext, Paris (Metal Ions in Biology and
Medicine, vol 4), indexed in Current Contents.
REGISTRATION FEE
The registration fee for active participant will be 40.000 Spanish pesetas (approximately US$ 300)
including the Proceedings Book, coffee-breaks, social events and Congress Banquet.
ACCOMODATION
Accomodation may be reserved on the registration form which will be distributed together with the
Second announcement to be published in May 1995.
SECRETARIAT
Dr. Mercedes Gomez
Laboratory of Toxicology and Biochemistry
School of Medicine
c/San Lorenzo 21
43201 Reus, Spain
Telephone: + 34-77-759376 FAX: + 34-77-759322
192

				

